# Regular Exercise Enhances Task-Based Industriousness in Laboratory Rats

**DOI:** 10.1371/journal.pone.0129831

**Published:** 2015-06-17

**Authors:** Nicholas C. Laurence, Lisa G. Labuschagne, Brent G. Lura, Kristin L. Hillman

**Affiliations:** Department of Psychology, University of Otago, Dunedin, New Zealand; Brock University, CANADA

## Abstract

Individuals vary greatly in their willingness to select and persist in effortful tasks, even when high-effort will knowingly result in high-reward. Individuals who select and successively complete effortful, goal-directed tasks can be described as industrious. Trying to increase one’s industriousness is desirable from a productivity standpoint, yet intrinsically challenging given that effort expenditure is generally aversive. Here we show that in laboratory rats, a basic physical exercise regimen (20 min/day, five days/week) is sufficient to increase industriousness across a battery of subsequent testing tasks. Exercised rats outperformed their non-exercised counterparts in tasks designed to tax effort expenditure, strategic decision-making, problem solving and persistence. These increases in performance led to quicker reward obtainment and greater reward gain over time, and could not be accounted for simply by increased locomotor activity. Our results suggest that a basic exercise regimen can enhance effortful goal-directed behaviour in goal-directed tasks, which highlights a potential productivity benefit of staying physically active.

## Introduction

Industriousness can be described as the behaviour of diligently exerting effort to complete goal-directed tasks. This behavioural tendency is variably described in the literature as grit, achievement orientation, persistence, and perseverance [1–5], and is classified within the five-factor personality model as a main facet of Conscientiousness [6–8]. To study industriousness in the laboratory, the behaviour can be operationally defined as selection of and persistence in goal-directed tasks, which results in maximal goal achievement for the individual. Given that industrious individuals exhibit higher task completion rates as compared to their less industrious peers, this facet of Conscientiousness is of interest in industrial/organisational psychology [9, 10], economic analysis [11] and as a predictive indicator of academic achievement [12]. Interventions that increase industriousness thus appeal to the individual and society.

For most individuals, effortful courses of action are aversive, and hence trying to increase industriousness is intrinsically challenging. Physical effort and mental effort, and the time associated with each, discount the perceived utility of a given course of action, making actions which require minimal effort more appealing [13, 14]. Aversion to effortful courses of action can be reduced by manipulating incentive value, either in terms of the distal outcome (high reward upon completion) or in terms of offering small proximal rewards along the way. The latter may involve tangible rewards or things such as positive feedback, verbal praise towards the action, or positive affective interludes [15–20]. Higher level cognitive tactics—such as reattribution training—can also increase persistence in difficult courses of action [21, 22], as can parsing one large effort-laden goal into smaller more immediately achievable goals [23]. All of these tactics, however, are used to prompt effortful behaviour only in direct relation to the current course of action. Thus industriousness is only improved for the particular task at hand, not necessarily across tasks.

One way that effortful goal-directed behaviour can be improved across tasks is via behavioural conditioning. Partial reinforcement schedules have long demonstrated that animals can be conditioned to expend more effort in instrumental tasks if they are intermittently rewarded, as opposed to continuously rewarded [24]. Conditioned effortful behaviour exhibits transfer effects across tasks [25–27] and across reward types [28], even if periods of continuous reinforcement are interleaved [29, 30]. Likewise if animals are repeatedly exposed to high-effort training scenarios, they are more likely to select high-effort options in the future (for review, see [2]), even if low- or no-effort options are available [31, 32]. High-effort trained animals are also more likely to persist in subsequent goal-directed tasks, demonstrating a type of ‘learned persistence’ that has been compared to established models of learned helplessness [15, 33, 34].

One explanation behind this high-effort conditioning effect is provided by Eisenberger (1992) in his learned industriousness model [2], wherein he suggests that by pairing subjectively effortful experiences with reinforcers, the sensation of effort might itself assume secondary reward properties over time (pg. 250). Learning that high-effort action leads to reward should then elicit approach behaviours towards high-effort options in subsequent choice scenarios. Alternatively or perhaps complementarily, repeated completion of effortful actions may lead to a diminished perception of subjective effort over time, reducing the normal discounting effect of effort. Successful completion of an effortful action could also increase belief/confidence that a second effortful pursuit will be successful, thus prompting engagement in high-effort actions (e.g. [35]). No matter the underlying mechanism, rewarded high-effort behavioural training in the laboratory—*e*.*g*. lever pressing, shuttle running, or solving anagrams—can successfully condition subjects to select high-effort options in other subsequent laboratory tasks.

A simple form of high-effort behavioural training that can be easily undertaken outside the laboratory environment is physical exercise. The cognitive benefits of physical exercise are being increasingly recognized (for review, see [36]), and correlational studies are progressively linking regular physical activity with higher academic achievement in children [37–39] and with improved job performance in adults [40–42]. While established exercise-mediated improvements in learning and memory likely contribute towards these positive outcomes in school and employment, an additional interpretation of the exercise-achievement relationship is that regular exercise may serve to improve industriousness. The subjective effort of exercise, if repeatedly experienced, could exert a transfer effect and prompt individuals to expend more effort in school- or job-related tasks, which would foster success in those domains.

The goal of this study was to use an animal model to test the hypothesis that regular exercise can enhance effortful goal-directed behaviour in subsequent testing tasks, thereby demonstrating an overall enhancement in industriousness. We operationally defined industriousness as the selection of and persistence in high-effort actions, resulting in higher net reward over time for the animal. This study proffers causality, in that the exercise regimen began weeks prior to the task testing period, and exercise was the only between group variable in question. By utilizing non-human subjects, this study also removes a number of significant confounds seen in the human exercise-achievement correlation, such as family environment, socioeconomic context and personality traits [42–44]. We found that rats who received regular exercise (20 min/day, 5 days/week) significantly outperformed control rats in a variety of testing tasks, resulting in higher net reward gain over time.

## Methods

### Subjects

**Fig 1:**
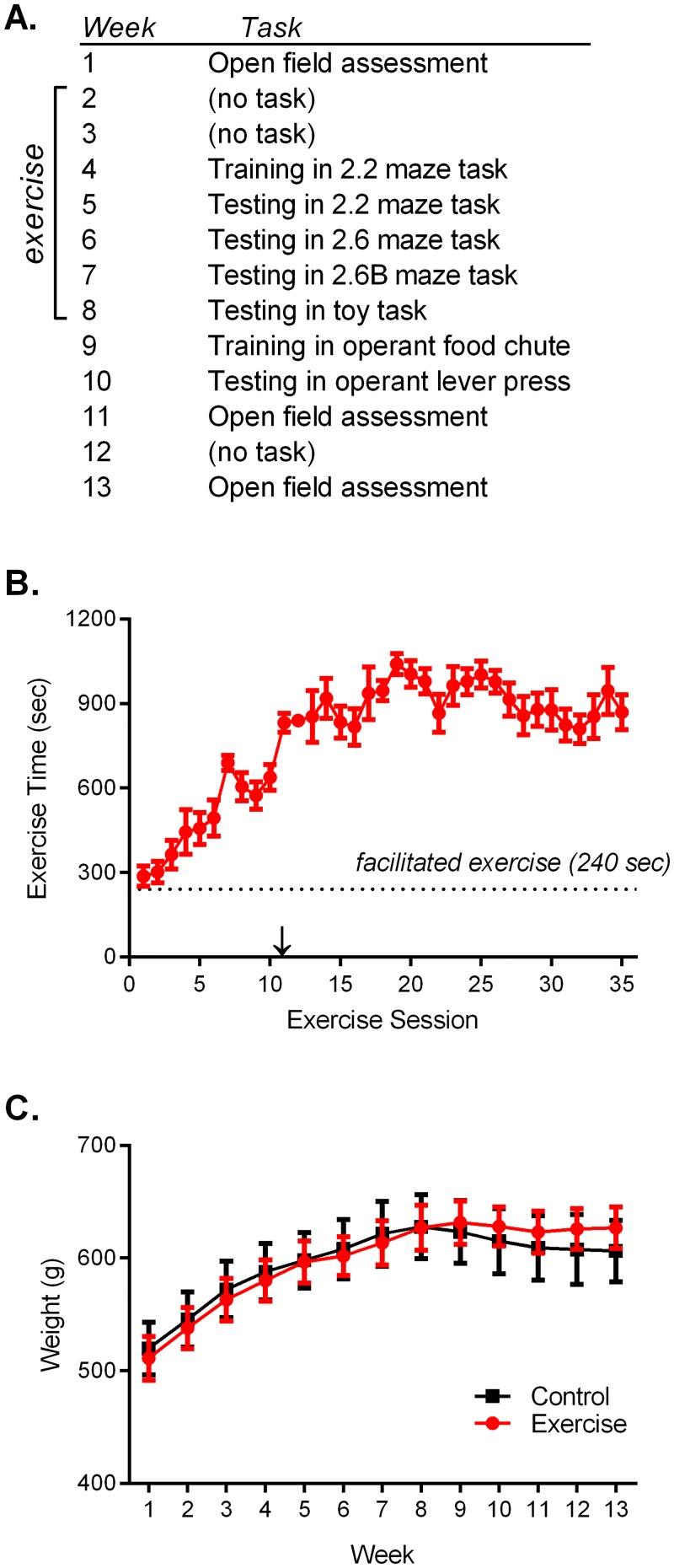
Thirteen Week Study. A) Outline of the study. In Weeks 2–8, Exercise rats were placed in a rodent running ball for five 20 min sessions/week. B) Each 20 min exercise session involved 4 min of facilitated running and 16 min of voluntary, free running. An arrow indicates the onset of testing tasks in Week 3. C) Individually calculated food rations ensured that body weights remained similar between groups. Data are shown as mean ± SEM; for each group *n* = 8.

Sixteen male Sprague-Dawley rats (450–600 g, 9–12 months in age, sourced from Hercus-Taieri) were pair caged for the 13 week study, which is outlined in Fig 1A. Rats were experimentally naive, and housed under a 12 hour light cycle. Experiments occurred at the beginning of the light phase, and exercise periods occurred towards the end of the light phase. Rats were food deprived to no less than 85% of their baseline weight to promote interest in food reward. Dried pineapple chunks, cocoa pop chocolate cereal and sweetened condensed milk were used as food reward in the testing tasks. In the two weeks preceding the start of the study, the rats were handled daily and habituated to the continuous T maze and the rodent running balls (fixed in place). All testing tasks used in the study were completely novel to the rats. Other than the pre-study apparatus habituations, the rats received only the training that is listed in Fig 1A, which occurred in the week immediately prior to the testing phase of the related task.

### Ethics Statement

All experiments were pre-approved by the University of Otago Animal Ethics Committee (protocol approval AEC 38/12), which adheres to international standards of animal welfare.

### Exercise Regimen

For a period of seven weeks, Exercise rats were individually placed in a rodent running ball (28 cm Critter Roller) for five sessions per week (20 min/session). Exercise behaviour was shaped with two 2-minute periods of assisted exercise during each session (from 0–2 min and 10–12 min), so that during each 20 min session the rat experienced four minutes of forced running and 16 minutes of free running. All rats increased their time spent free running week-by-week (Fig 1B; r^2^ 0.37, slope 15.6±1.2, p<0.001). While the Exercise rats were running, Control rats were placed in a 33x30x28 cm open field containing an identical rodent running ball, which was fixed in place. Most of the Control rats spent the majority of the 20 min session seated inside the stationary ball, although they were free to travel between the open field and stationary ball. Careful monitoring of animal weight, paired with daily adjustment of food rations, ensured that no significant weight differences developed between the groups throughout the study (Fig 1C; Group F(1,14) = 0.01, p = 0.99). Both groups gained weight throughout the study (Time F(11,154) = 54.8, p<0.001).

### Open Field Assessment

**Fig 2:**
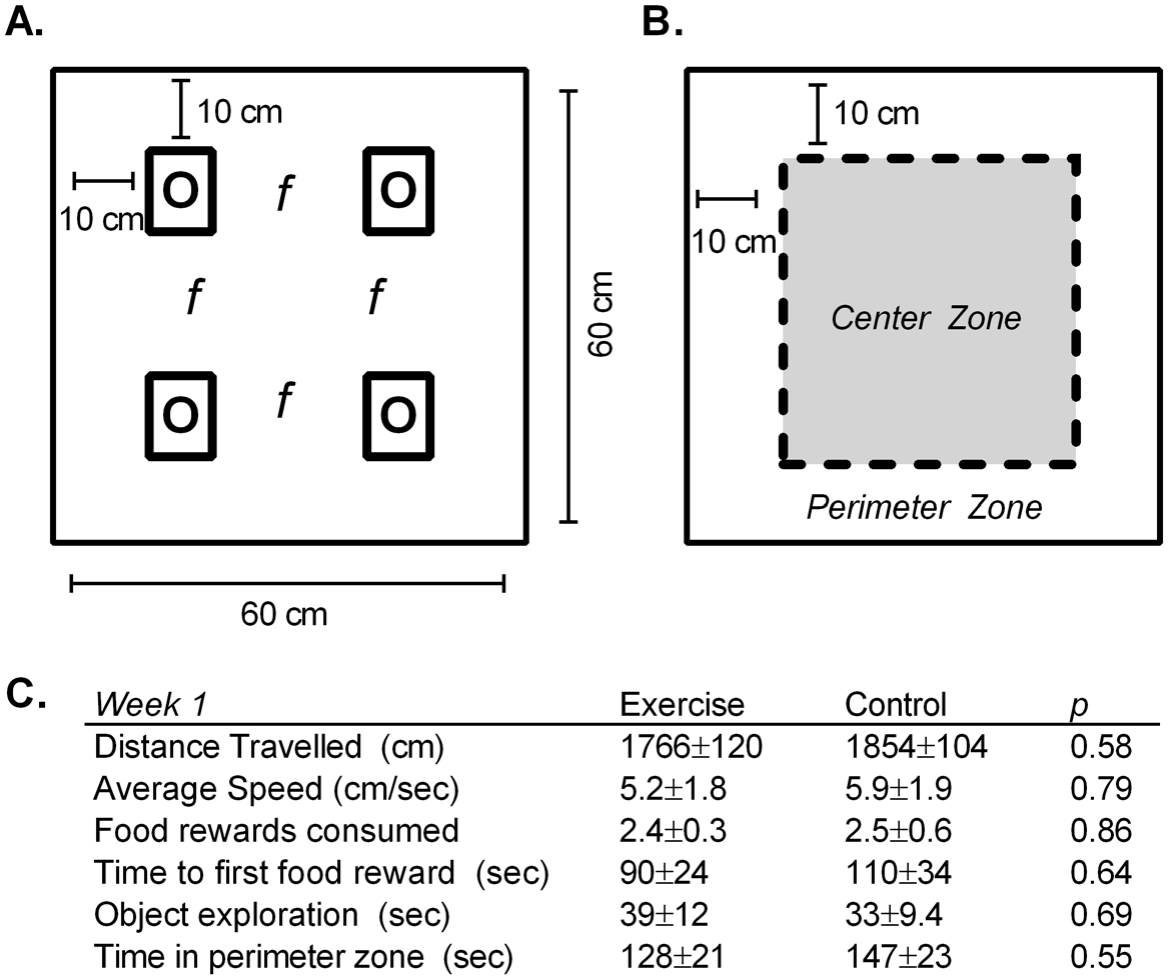
Open Field Assessment. A) Schematic of open field arena indicating placement of four novel objects (O) and four food rewards (*f*). B) Schematic of open field arena indicating 40x40 cm square center zone excluded for assessment of thigmotaxis. C) Data from Week 1, indicating no differences between groups in any of the measures. Data are shown as mean ± SEM; for each group *n* = 8.

The Exercise and Control groups had been allocated based on three days of open field assessment (5 min/day) conducted in Week 1, prior to the start of the exercise regimen. Rats were put in a 60x60x22cm open field containing four novel objects (small toys changed daily) and four food rewards (Kellogg’s Froot Loops), as illustrated in Fig 2A. Novel objects and food rewards were used to assess exploratory behaviour and food motivation. For each session, EthoVision tracking software was used to calculate the following: distance travelled, run speed, time spent in center/perimeter zones (Fig 2B), time to first food reward, and time spent exploring novel objects. The arena was cleaned with disinfectant (ClearKens TEGO 2000) between animals. Experimental groups (Exercise and Control, for each *n* = 8) were then assigned based on a counterbalance of baseline locomotor activity (Fig 2C). Three days of identical open field assessment (5 min/day) were conducted at the end of the testing task phase to assess end-of-study activity patterns in Weeks 11 and 13.

### Testing Tasks

Beginning four weeks into the exercise regimen, the rats were tested on a series of tasks (described below) designed to tax effort expenditure, decision-making, problem-solving, and effortful persistence, all key components of industriousness [6–8].

#### 1) Maze Tasks

**Fig 3:**
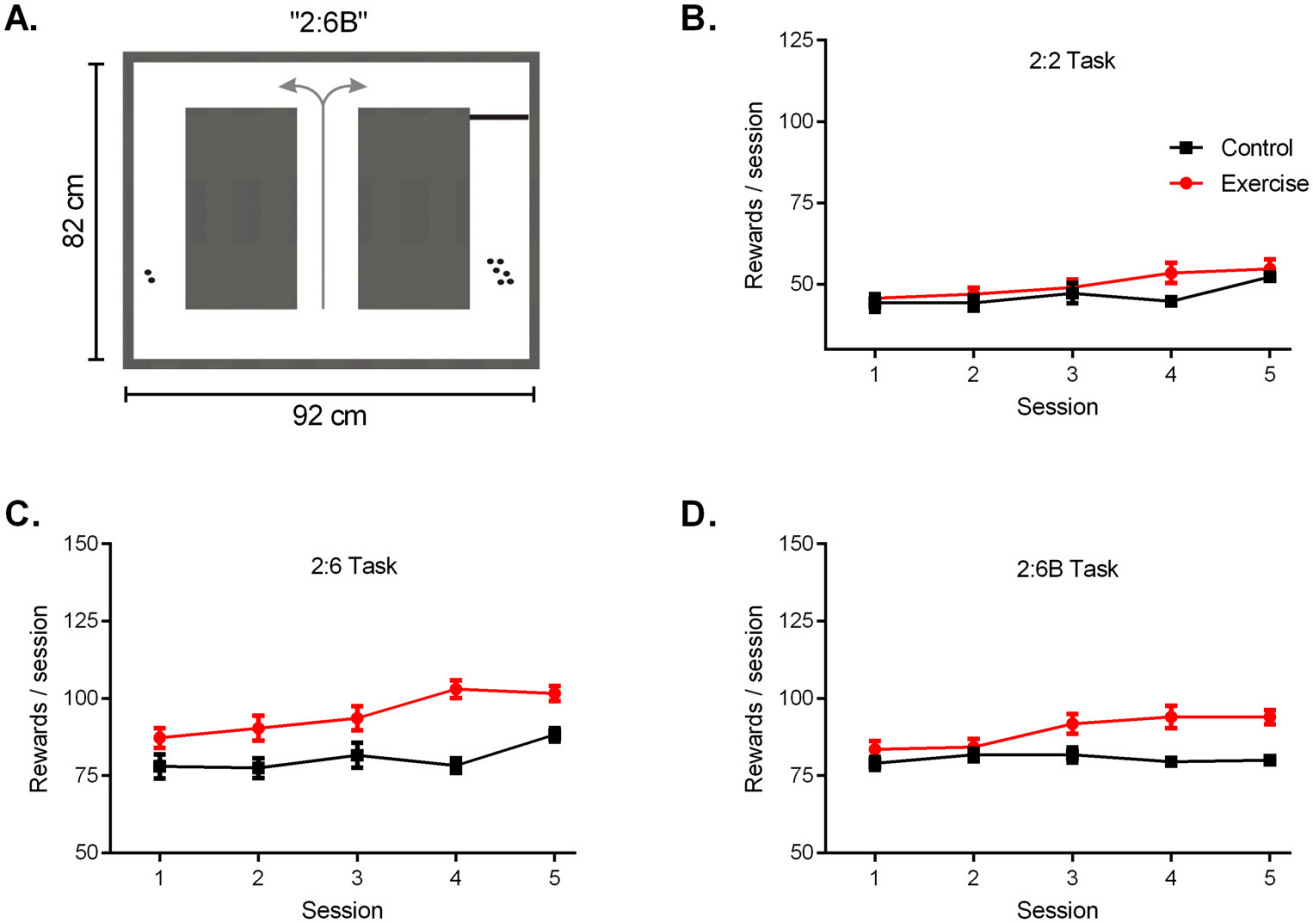
Maze Tasks. A) Schematic of the continuous T-maze, illustrating the 2:6B configuration. On each trial, the rat would run up the mid-stem (indicated by arrows) and proceed down the left or right arm. Each arm was baited with food pellets (indicated by dots), in either a 2:2 or 2:6 ratio. A bold line indicates where a climbing barrier was placed to create the 2:6B configuration. B) When given 5 min in the 2:2 configuration, there was no difference between groups in reward gain across sessions. C) When given 20 trials in the 2:6 configuration, the Exercise group selected the HR option more often than Controls, resulting in greater reward gain. D) When given 20 trials in the 2:6B configuration, the Exercise group selected the HEHR option more often than the Controls in the later sessions, resulting in greater reward gain. Data are shown as mean ± SEM; for each group *n* = 8.

Rats were individually placed into the central stem of a continuous T-maze measuring 82x92x25 cm. For each trial, the rat had to run up the mid-stem, turn right or left at the junction, and proceed unidirectionally towards the food reward (see Fig 3A). Trials were run continuously at a self-set pace; any attempts to reverse direction or bypass the mid-stem were blocked by the experimenter. An initial week of training (10 min/day for five days) occurred where two cereal pellets were placed at each reward site (making for a 2:2 reward ratio) and the rats progressively learned to run in a unidirectional manner via correction by the experimenter. By the end of the training week, all rats achieved the minimum criterion of 20 unidirectional trials in 10 min (data not shown).

In the first week of maze task testing, each rat was tested in the 2:2 configuration. During each session (5 min/day for five days), the rat could run as many trials as desired to gain food reward. The purpose of this 2.2 Task was to assess self-directed repetition of a rewarded course of action. Rats who ran trials diligently for the whole of the five minutes would complete more trials, thereby earning greater reward, as compared to rats who ran slowly or intermittently.

In the second week of maze task testing, the rats were tested in a 2:6 configuration, where two cereal pellets were placed in one arm of the maze and six cereal pellets placed in the other, creating a low-reward/high-reward (LR/HR) scenario. The HR side was counterbalanced across groups, and each rat given 20 trials/day for five days. The purpose of this 2.6 Task was to assess strategic decision-making. When unconfined by time, but still limited to a set number of trials per day, exploitation of the HR choice option would maximize reward gain.

In the third week of maze task testing, the rats were tested in a 2:6B configuration. In this 2:6B configuration, a 20 cm climbing barrier (‘B’) was placed in front of the six cereal pellet site, requiring the rat to invest effort and climb the barrier to reach the HR. This created a low-effort LR/high-effort HR (LELR/HEHR) scenario. Again the HEHR side was counterbalanced across groups, and each rat given 20 trials/day for five days. The purpose of this 2.6B Task was to assess cost-benefit decision-making. Again when unconfined by time, but still limited to a set number of trials per day, exploitation of the HR choice option would maximize reward gain, but also require extra effort.

#### 2) Toy Task

Each rat was placed in a 38x27x19 cm test cage which contained a rubber pet toy (9x2 cm PetSmart Chaser). The ovoid toy had slits on the surface, and was loaded prior to each session with three pineapple chunks as food reward. Rats were given 10 min/day for five days to try and remove food reward from the toy, which required dexterous manipulation of the toy with forelimbs and mouth, and/or rolling and leveraging the toy to extract the fruit. When there was physical contact between forelimb-toy or nose/mouth-toy within the 10 minutes, this was coded as ‘time spent working to remove food’; when there was no rat-toy contact or when there was non-directed rat-toy contact, this was coded as ‘time not working.’ The latter was sub-scored with regard to time spent eating food reward, time spent grooming, and time spent rearing (data not shown). The purpose of the Toy Task was to assess effortful persistence and problem-solving. Maximal productivity (rewards gained over set period of time) required cognitive effort and physical effort to deduce and repeat an efficient food extraction technique.

#### 3) Operant Box Task

Rats were individually placed in a 24x20x23 cm standard operant box. Each box housed two extendable levers and a food hopper, in which a dipper delivered sweetened condensed milk as food reward. In the first week (training week), the rats were trained to put their head in the food hopper to receive food reward. During this training period the levers were not extended, and food reward was automatically delivered in a pattern of three seconds available, three seconds unavailable. The hopper was illuminated each time food reward was available. Each rat was given one 10 min session/day, for five days, to reach a criterion of 50 hopper entries/session. Most rats achieved this criterion by day two; all rats achieved this by day four (data not shown).

In the second week (testing week), both levers were extended and lever pressing was required for reward delivery. Since the rats had not been trained on a lever pressing paradigm previously, this meant that in order to obtain food reward, the rats needed to explore the levers, press the levers, and progressively build an association between lever pressing and food reward delivery. Each rat was given one 15 min session/day, for seven days, in the apparatus. Behavioural output was categorized into four stages: No Association, Discovery, Establishment, and Exploitation. *Discovering* the association between lever pressing and reward was set as: within one session, ≥5 lever presses that were followed by a hopper entry within the next 10 sec (even if entry occurred after the three second reward availability period). *Establishment* of the association between lever pressing and reward was set as: within one session, ≥10 lever presses that were followed immediately by a hopper entry within the three second reward availability period. *Exploitation* of the lever pressing strategy was set as: within one session, ≥20 lever presses that were immediately followed by a hopper entry within the three second reward availability period. The purpose of the Operant Box Task was to assess effortful persistence and problem-solving. Again, maximal productivity (rewards gained over set period of time) required cognitive effort and physical effort to deduce (discover and establish) and persistently repeat (exploit) the lever press technique.

### Data Analysis

All data were analyzed using GraphPad Prism 5.01. Group differences were determined using a one- or two-way ANOVA, depending on number of factors, with post-hoc Tukey’s multiple comparisons test. Pairwise comparisons of overall reward gain and open field measures were assessed by unpaired t-test. Linear regressions were performed to test for linear trend across time. For all analyses, significance was assumed at p<0.05. In figures, significance is denoted as follows: *p<0.05, **p<0.01, ***p<0.001.

## Results

### Maze Tasks

In the maze tasks, the rats were allowed to run unidirectionally in a continuous T-maze to obtain food reward; the 2:6B configuration is illustrated in Fig 3A. The rats ran at a self-set pace, with sessions comprised of either five minutes (2:2 task) or 20 trials (2:6 and 2:6B tasks). High-reward locations were counterbalanced between and within animals. In the initial 2:2 configuration, the rats were allocated 5 min/session to complete as many trials as desired within the timeframe. Across sessions, the Exercise rats ran a mean of 32±1.2 trials per session, while the Control rats ran 29±2.6 trials per session (p = 0.29, t-test). In terms of overall reward gain, there was no significant difference between groups (Fig 3B; Group F(1,14) = 1.84, p = 0.20, Time F(4,56) = 7.1, p<0.01).

In the 2:6 configuration, the rats were limited to a set number of 20 trials/session. The Exercise group exhibited a higher choice percentage for the HR side, resulting in higher net reward as compared to the Control group (Fig 3C; Group F(1,14) = 14.97, p = 0.002, Time F(4,56) = 10.75, p<0.0001). In the 2:6B configuration, a climbing barrier was inserted prior to the high-reward, creating an HEHR scenario. The rats were again allocated a set number of 20 trials/session. In the 2:6B configuration, the Exercise group had a higher choice percentage for the HEHR option, resulting in higher net reward as compared to the Control group (Fig 3D; Group F(1,14) = 11.24, p = 0.005, Time F(4,56) = 4.19, p = 0.005). Trend analyses showed positive linear trends in reward gain by the Exercise group across sessions in the 2:2 configuration (r^2^ 0.95, slope 2.4±0.3, p = 0.005), the 2:6 configuration (r^2^ 0.89, slope 4.2±0.81, p = 0.015), and the 2:6B configuration (r^2^ 0.87, slope 3.08±0.7, p = 0.022). Trend analyses of Control group data showed no significant linear trends in any of the three configurations. Across the trial-limited 2.6 and 2.6B sessions, the Exercise rats took 376±29 sec per session, while the Control rats took 316±18 sec per session (p = 0.08, t-test).

### Toy Task

In the toy task, the rats were placed in test cages containing an ovoid rubber toy loaded daily with three pieces of dried fruit reward. The goal of the task was to remove as many of the three food rewards within the 10 min session, which required manipulating the toy either via small scale dextrous manoeuvres or larger scale rolling manoeuvres.

**Fig 4:**
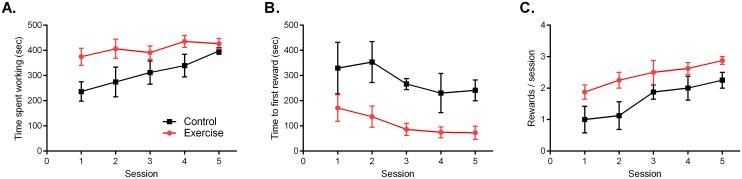
Toy Task. A) Across the five 10-minute sessions, the Exercise group spent more time working to remove food reward as compared to Control. B) The Exercise rats were quicker to obtain their first reward and (C) obtained more rewards over the course of the five day testing period as compared to Control. Data are shown as mean ± SEM; for each group *n* = 8.

While both groups showed initial interest in the toy and its contents, the Exercise rats spent more overall time working to remove the food reward as compared to Controls (Fig 4A; Group F(1,14) = 5.06, p = 0.04; Time F(4,56) = 5.83, p = 0.001). The Exercise rats were also quicker to obtain their first food reward each day as compared to Controls (Fig 4B; Group F(1,14) = 7.22, p = 0.02; Time F(4,56) = 3.62, p = 0.01). The Exercise rats achieved higher reward gain over the five day period (Fig 4C; Group F(1,14) = 4.82, p = 0.04; Time F(4,56) = 10.9, p<0.0001). A positive linear trend in time spent working was observed in the Control group (r^2^ 0.98, slope 38.8±2.6, p<0.001), while a negative linear trend in time to first reward was observed in the Exercise group (r^2^ 0.88, slope -25.9.8±5.6, p<0.001). Both groups exhibited a positive linear trend in reward gain across sessions (Exercise r^2^ 0.97, slope 0.24±0.02, p = 0.002; Control r^2^ 0.93, slope 0.34±0.06, p = 0.009).

### Operant Box Task

In the operant box task, the rats were placed in standard operant boxes and challenged to discover and exploit the association between lever pressing and food delivery. All rats had been previously shaped—via one week of initial training—to place their head in the food chute to obtain food reward. During this initial training phase no levers were available, and food was delivered automatically in a pattern of three seconds available, three seconds unavailable.

**Fig 5:**
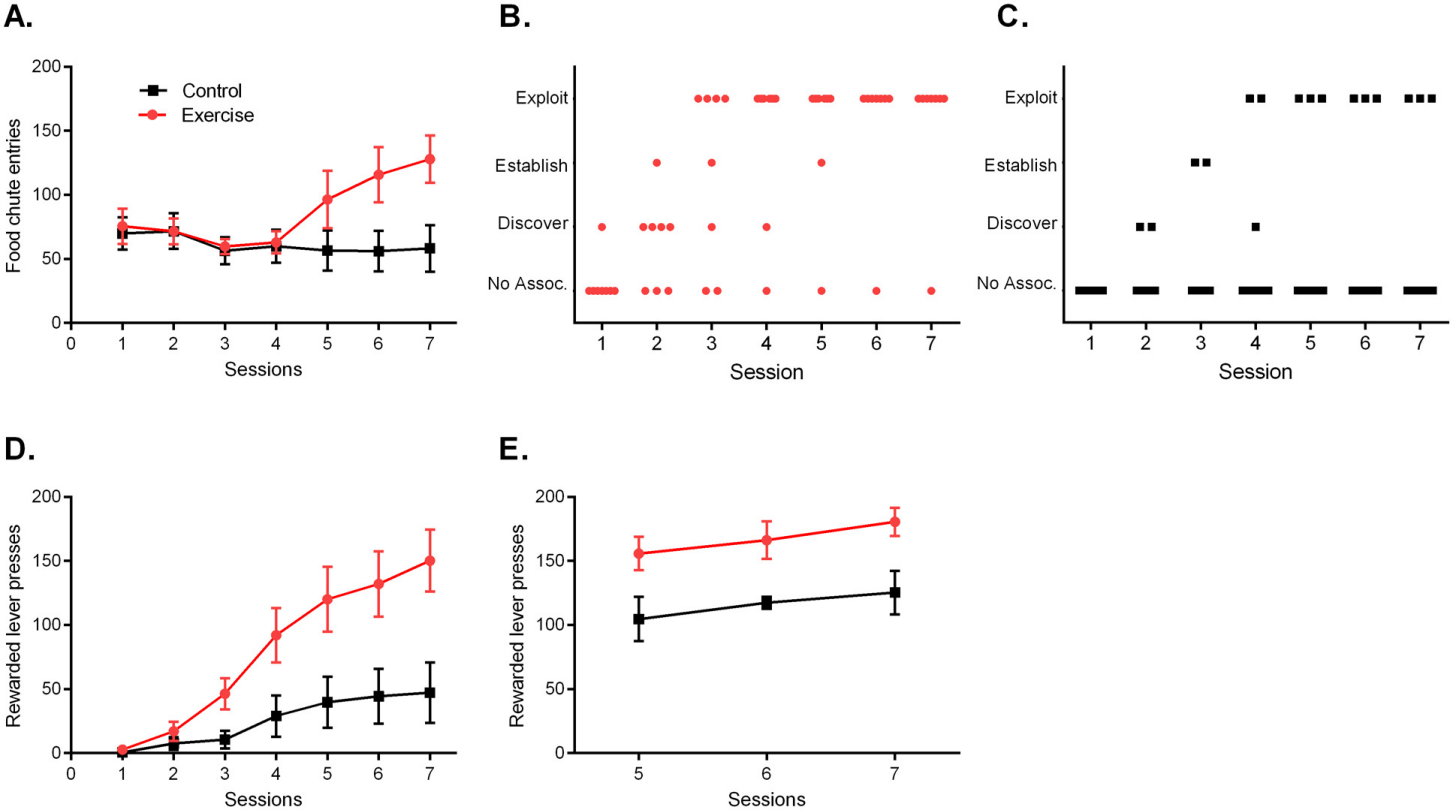
Operant Box Task. A) When given 15 min in the novel, levered scenario, both groups made multiple entries into the food chute, suggesting knowledge of reward location. B) Stage progression for the Exercise group; each rat is indicated as a dot for each session. C) Stage progression for the Control group; each rat is indicated as a square for each session. D) Across sessions, the Exercise group procured significantly more reward as compared to Control group. E) When comparing only rats who reached the exploitation stage for three consecutive sessions (n = 6 Exercise, n = 3 Control), the Exercise exploiters achieved more lever presses, and therefore more reward, per session. Data are shown as mean ± SEM; for each group in panels A-D, *n* = 8.

During the testing sessions, food was only made available after a lever press. For maximal reward gain, rats needed to master three stages: rats needed to *discover* the association between lever pressing and reward; *establish* the association; and then *exploit* the action (see Methods for each stage’s criterion). All rats made numerous entries into the food chute in each testing session, suggesting remembrance of reward location and motivation to eat (Fig 5A; Group F(1,14) = 2.76, p = 0.12; Time F(6,84) = 2.5, p = 0.03; Group x Time F(6,84) = 3.6, p = 0.003). Over the course of seven sessions (one session/day), 11 of the 16 rats successfully discovered, established and exploited the relationship between lever pressing and food delivery. Seven of the eight Exercise rats, and three of the eight Control rats, reached the exploitation stage (Fig 5B and 5C). This was not a significant proportional difference between groups in mastery (p = 0.12, Fisher’s Exact Test), however the Exercise group achieved more reward across sessions (Fig 5D; Group F(1,14) = 7.6, p = 0.02; Time F(6,84) = 23.6, p<0.001; Group x Time F(6,84) = 6.08, p<0.001).

To determine if rate of lever pressing differed between groups once animals reached the exploitation stage, the final three sessions (session 5, 6 and 7) were specifically examined. The data set was constrained to only rats who had been in the exploitation stage for all three of those sessions (n = 6 Exercise, n = 3 Control). Here, the Exercise group demonstrated significantly more lever presses per session as compared to the Controls (Fig 5E; Group F(1,21) = 17.9, p<0.001).

### Overall Productivity

**Fig 6:**
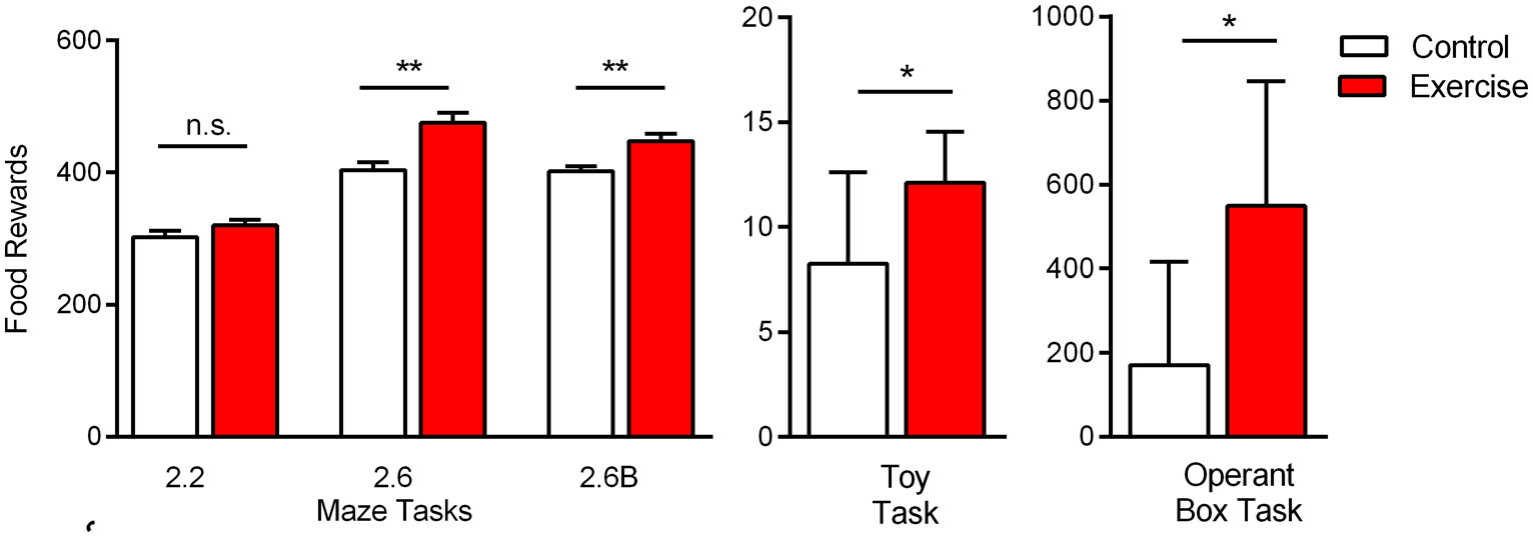
Cumulative Reward Gain. When each group member’s overall reward gain was pooled into a measure of cumulative reward gain for the group, the Exercise group obtained more reward at the end of the study as compared to Control. Significant pairwise differences (t-test, p<0.05) were observed in all tasks except the 2:2 maze task. Data are shown as mean ± SEM; for each group *n* = 8.

Each group member’s overall productivity during the study was quantified (total number of rewards gained over the six week testing period), and pooled into a group total. The Exercise group obtained significantly more food reward as compared to the Control group (Fig 6; Group F(1,70) = 10.8, p = 0.002). On the individual tasks, differences in reward gain (p<0.05, t-test) were observed between groups for all except the 2:2 maze task. When examined as net productivity over the six week task period, the Exercise group earned 14,593 of the total available rewards (c. 29,420), while the Control group earned 10,643 (χ^2^ (1) = 1082, p<0.001,).

### Open Field Assessment

**Fig 7:**
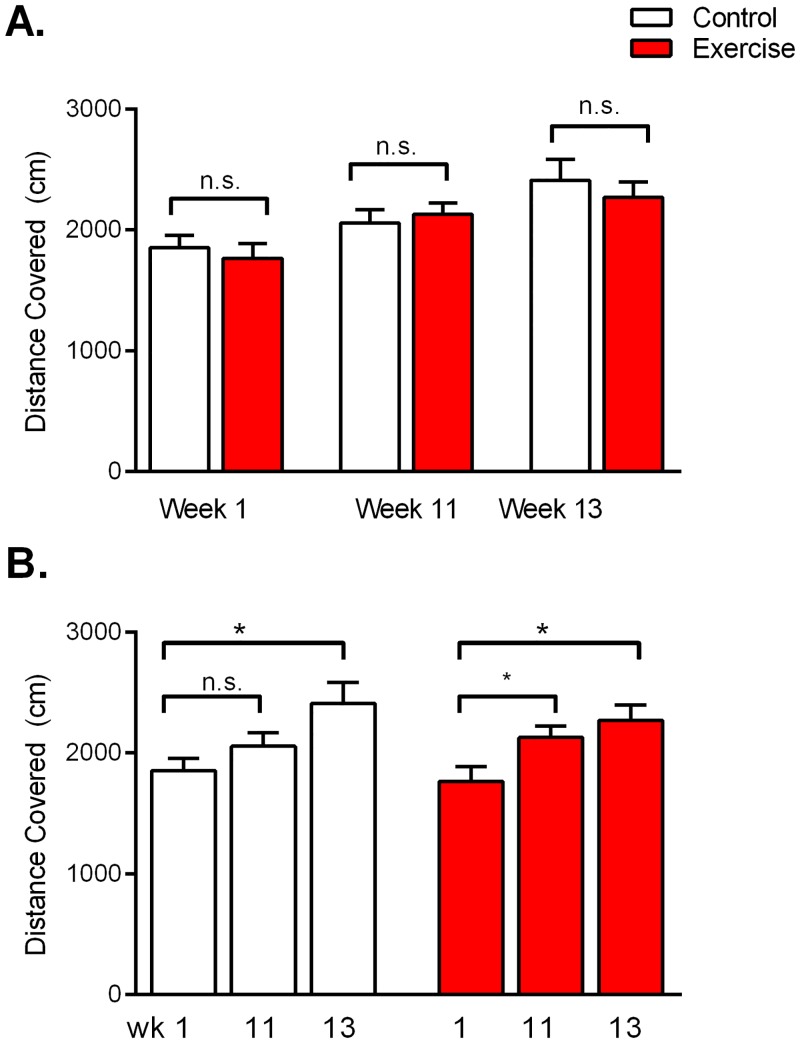
Locomotor Activity in Open Field Assessment. A) In each week of assessment, no differences in distance travelled were observed between the groups, both in weekly pairwise comparisons (t-test, α 0.05) and overall assessment (ANOVA, α 0.05). B) Within group comparisons of the data in (A) revealed that each group increased its distance travelled over the course of the 13 week study. Data are shown as mean ± SEM; for each group *n* = 8.

Open field assessments of locomotor activity were conducted in Weeks 1, 11 and 13 of the study. Data from Week 1 were used to counterbalance group assignments in terms of baseline motor activity, prior to the exercise regimen starting in Week 2 (see Fig 2C). Data from Weeks 11 and 13 were used to assess any end-of-study changes between groups in locomotor activity, exploratory behaviour or food motivation; no significant differences were found (Table 1). Across the three open field assessment periods (Weeks 1, 11 and 13), both groups increased their exploration distances, however there was no overall difference between groups (Fig 7A; Group F(1,42) = 0.26, p = 0.62; Time F(2,42) = 9.1, p<0.001). Within the groups, each group was significantly more active in open field exploration in Week 13 as compared to Week 1 (Exercise F(2,46) = 4.5, p = 0.02; Control F(2,46) = 4.0, p = 0.04; post-hoc Tukey’s).

**Table 1:** Open Field Assessment Measures from Weeks 11 and 13.

		*Week 11*			*Week 13*	
	Exercise	Control	*p*	Exercise	Control	*p*
Distance travelled (cm)	2130±94	2056±112	0.62	2270±130	2411±173	0.52
Average speed (cm/sec)	5.9±2.1	5.6±2.4	0.92	7.1±2.3	8.4±3.6	0.77
Food rewards consumed	4.0±0	4.0±0	—	4.0±0	4.0±0	—
Time to first food reward (sec)	26±5.7	11±5.9	0.09	11±2.2	10±4.1	0.83
Object exploration (sec)	46±6.4	61±11	0.26	67±13	51±21	0.53
Time in perimeter zone (sec)	120±29	89±20	0.39	81±17	79±20	0.94

## Discussion

### Experimental design for studies of industriousness

For this study we have operationally defined industriousness as selection of and persistence in goal-directed tasks, resulting in maximal goal achievement for the individual. Selection of goal-directed tasks can be seen in our tasks simply via task engagement. In all tasks, the rat could choose to engage in the task at hand, or alternatively groom, sleep, or explore the environment. Voluntary task engagement is important, as industriousness is often viewed as largely self-directed behaviour; tasks completed by force or under duress are often excluded from discussions of human industriousness [45–47].

While selecting to engage in a task is key, it alone is not a sufficient indicator of industriousness, as evidenced if a subject fatigues/forfeits halfway through the task. Likewise indicators of task learning and memory are not necessarily indicative of industriousness, as two subjects may equally learn and remember how to perform a task, but then differ in their willingness to volitionally repeat the task once learned. An individual must select *and persist* in the task to the point of completion, and to a point of maximal gain within a limited timeframe. Industriousness thus demands extra effort, via persistence, problem-solving, strategic effort allocation and diligent repetition [45]. In real-world productivity situations, sometimes one must work quickly to earn the most reward in a task, while other times one must work strategically; almost always there is a set timeframe in which to complete the task-at-hand. The panel of tasks used in this study was designed to mimic these challenges.

Maximal reward gain in the time-limited 2.2 maze task required continual running for the 5 min period, while maximal reward gain in the trial-limited 2.6 and 2.6B maze required a level of problem solving. Hence we used a time limit in the 2.2 task to tax self-directed persistence and diligent repetition (working quickly), and a trial limit in the 2.6 and 2.6B tasks to assess cost-benefit analysis and effort allocation (working strategically). Each task was run for only five days (set timeframe). In the 300 seconds of the 2.2 task, both groups completed a similar number of trials, resulting in no difference in overall reward gain (Fig 6). In the 20 trials of the 2.6 and 2.6B tasks, the Exercise group earned more reward in both configurations (Fig 6) due to higher selection of the HR six pellet option (Fig 3C and 3D). The time needed to consume the HR contributed towards a longer—though not significantly different—time per session for the Exercise group. Taken together the Exercise rats appear to work marginally quicker, but notably more strategically, as compared to their Control counterparts in the maze tasks.

The toy task and operant box task each required problem-solving, persistence and then diligent repetition for maximal reward gain within a set timeframe. In this study the operant box was used in a non-traditional way (i.e., with very limited shaping and for a set period of only seven days); again this was intended to mimic real-world productivity challenges, where a subject may be presented with an unfamiliar situation and challenged to deduce and exploit the appropriate action within a restricted timeframe. We felt the non-traditional use of the operant box herein appropriately assessed problem solving, persistence and diligent repetition, but certainly future studies could thoroughly investigate operant behaviour between groups in terms of response ratios or time needed for mastery. Doubling the session number for the operant task—and indeed for all tasks herein—would provide an informative indication of the trajectory of mastery between groups. However for this study we purposefully imposed ‘deadlines’, as industriousness is often about efficient completion of tasks [6], rather than prolonged persistence in a task unrestricted by time or number of attempts [3]. In the operant task, individuals from the Exercise group were quicker to deduce and exploit the lever press (Fig 5B and 5C), as well as diligently repeat the action at a higher rate as compared to Controls (Fig 5E), resulting in a higher reward gain at the end of the testing week (Fig 6).

The toy task was also intended to mimic a real-world productivity challenge, necessitating trial-and-error and persistence for maximal goal achievement, within a restricted timeframe of five days. While the Exercise group achieved more reward throughout the task (Fig 6), they did not demonstrate visibly different tactics as compared to the Controls. Anecdotally, both groups trialled similar manoeuvres to remove the food reward, with successful extractions based more on strategic leverage and dexterity, rather than brute force. Thus physical strength did not appear to underlie the Exercise groups’ increased success in the toy task, rather the Exercise rats were quicker to master extraction techniques (Fig 4B) and worked more diligently throughout each 10 min session to extract the food pieces (Fig 4A).

Differences in physical strength should also be considered for barrier climbing in the 2.6B task, and locomotor activity in the operant box task (where heightened motor activity could increase the probability of pressing levers and discovering the lever-food association). The Exercise rats did select the barrier option more frequently in the 2.6B task (Fig 3D), and also discovered and exploited the lever-food association sooner (Fig 5). While we cannot rule out a difference in physical conditioning between groups, locomotion and speed levels did not differ between groups in the post-study open field assessment (Fig 7A and Table 1). Regardless, this highlights the need to always test combinations of physical effort and cognitive effort when studying industrious behaviour. The five tasks used in this study provide such a composite assessment, as maximal reward gain in many of the tasks demanded a degree of cognitive effort, in addition to physical effort.

### Exercise as a modulator of industriousness

Improving industriousness in human individuals or groups has long been an interest of industrial psychology. However, at the same time the propensity to work hard is often viewed as a personal attribute [48, 49] that remains relatively rigid in adulthood [50, 51], and thus attempting to improve individual industriousness in the adult workforce is counterintuitive. However our results here in laboratory rats, and the results of others in laboratory animals and in humans (for review, see [2]), increasingly suggest that the propensity to exert effort in tasks can be modified in adulthood.

One way effort expenditure can be increased in adult subjects is via use of psychostimulants, however net productivity may not increase if stimulant-driven effort is wasted on premature responding or directed towards high-risk endeavours [52, 53]. Effort expenditure can also be increased via behavioural training. In rodents for example, high-effort conditioning (e.g. reinforced shuttle running [25] or digging [33]), can increase subsequent selection and persistence in HEHR courses of action (for review, see [2]), which is more indicative of industriousness. While effective, this does require rewarding the high-effort training period with tangible rewards or other forms of external positive feedback.

The results presented herein identify a simple form of behavioural training—regular physical exercise—that is not explicitly rewarded, and yet able to significantly increase subsequent productivity in testing tasks. Of note, this effect extended beyond the exercise period itself, as daily exercise was stopped at the beginning of Week 9 yet between-group effects were observed in the operant box testing of Week 10. We chose to utilize one set period of exercise per day, versus continual access to running wheels in the home cage (e.g. [54]), as we felt this more accurately mirrored human exercise behaviour. Rodent running balls—versus running wheels—also proved a useful experimental tool in that they allow monitored exercise sessions of set durations, comprised of facilitated running, voluntary running, or a mixture of the two. Although not done in this study, an accelerometer could be fitted to the running balls to enable distance and speed measurements for future studies. We used older-than-average laboratory rats (9–12 months) to mirror middle-aged adulthood in humans, for whom occupational productivity benefits are traditionally of interest.

The lack of explicit reward in our effort-training condition (exercise) provides an important point of difference in comparison to previous conditioning studies of industriousness (e.g. [26, 27, 33]). High-effort training, that is then rewarded, would be expected to shift subsequent choice behaviour towards high-effort options, since high-effort is the response contingency that has been previously paired with reward [2, 32]. This pairing of HE-HR may underlie the positive linear trend across sessions we observed in many of our individual tasks, however it fails to explain why non-rewarded exercise training provides a transfer effect of enhanced effort expenditure across the composite of testing tasks.

One interpretation of our data is that perhaps regular exercise serves to decrease the subjective experience of physical effort intensity (i.e., motor actions that once felt ‘effortful’ no longer seem to be as energetically demanding due to improved physical fitness), however this does not fully address the Exercise rats’ improved performance in the problem-solving laden tasks, such as the operant box, the toy task and the 2:6 maze task. A second interpretation is that perhaps regular exercise alters the positive/negative valence of effort expenditure (i.e., activities that once seemed aversively ‘effortful’ are now approached as engaging or challenging). A shift in valence could stem from exercise-mediated improvements in mood; this has been reported in humans [55], likely linked to exercise-mediated changes in neurotransmitter and neuropeptide levels [56, 57]. While such alterations in perceptual assessments and mood cannot be assessed in our rodent study, this represents an important area for future investigation in human subjects.

A third interpretation is that perhaps regular exercise—by burning calories—increases the hunger levels of the Exercise rats, and subsequently increases motivation for the food reward in the testing tasks. We were aware of this potential confound from the start of the study, hence the animals’ weights were monitored closely and daily adjustments to food rations were made to ensure no bodyweight difference developed between the groups (Fig 1C). Data from the open field assessment in Weeks 11 and 13 suggest that food motivation was similar between groups, with no between group difference in Time to First Food Reward or Number of Food Rewards Consumed (Table 1). Nonetheless, repeating the study with tasks utilizing a non-food based goal would be insightful.

A fourth interpretation is that the physical activity regimen serves as a form of enrichment that drives improvements in learning and memory, which accounts for the Exercise rats’ enhanced performance in the subsequent testing tasks. Environmental enrichment for rodents is well-known to improve behavioural performance in tests of learning and memory, anxiety and depressive-like behaviours, and enhance mechanistic measures such as neurogenesis and cerebral blood flow (for review, see [58]). However improvements in learning and memory alone do not necessarily equate to enhanced productivity; being industrious requires learning and mastering a task, but also volitionally expending effort to repeat it once learned. The Exercise rats in this study may well be exhibiting a cognitive advantage stemming from an enrichment effect, but they also consistently expended more effort than the Control rats. For example they climbed the barrier more frequently in the 2.6B maze task (Fig 3D), they spent more time working to extract food in the toy task (Fig 4A), and they pressed the lever more frequently once they mastered the operant box task (Fig 5E). Whether the Exercise group’s enhanced industriousness is due to a component of intelligence (via general enrichment) or due to a component of effort (via a transfer effect) cannot be fully disentangled, but may not need to be, as synergy between the two components would arguably result in optimal industriousness.

Our results here in a rat model may provide important insight into the exercise-achievement correlation currently being explored in humans. As the cognitive benefits of exercise are being increasingly recognized (for review, see [36]), positive correlations between physical fitness and academic/occupational success are also emerging (e.g. [40–42, 59–61]). Physical activity therefore not only improves performance in a range of laboratory-based cognition tasks, but it also appears to provide a practical benefit of enhanced achievement at school/work. Our results would suggest that the latter may be in part driven by enhanced selection of and persistence in goal-directed tasks, resulting in a net increase in productivity. Indeed selection of effortful pursuits, and persistence in those challenges over time, is often a better predictor of achievement in school/work than measures such as IQ [1].

To date, interpretation of the exercise-achievement relationship in humans has been limited by its correlational nature. For example, subjects who exhibit high Conscientiousness at work/school are more likely to exhibit high Conscientiousness in relation to their personal health, and subjects who have high-paying jobs with more flexible hours are more likely to have free time to engage in regular exercise [42]. While intervention-based exercise studies (e.g. [59–61]) are starting to better address causality, additional human confounds still remain. For example, adherence to exercise regimens, biases in self-report measures, socioeconomic factors, and exercise-mediated changes in mood, self-esteem and/or self-efficacy can affect interpretation of the exercise-achievement relationship. By utilizing a non-human animal model in this study, we remove a number of these confounds, and demonstrate that a simple exercise regimen can causally increase industriousness in a series of goal-directed testing tasks.

## Supporting Information

S1 File Dataset for exercise-industriousness study. (XLSX)
